# The anticancer mechanisms of exopolysaccharide from *Weissella cibaria* D-2 on colorectal cancer via apoptosis induction

**DOI:** 10.1038/s41598-023-47943-7

**Published:** 2023-11-30

**Authors:** Yurong Du, Lei Liu, Weiliang Yan, Yang Li, Yuanzhe Li, Kang Cui, Pu Yu, Zhuoyu Gu, WanCun Zhang, Jianguo Feng, Zhen Li, Hao Tang, Yabing Du, Huan Zhao

**Affiliations:** 1 Oncology Department, The First Affiliated Hospital of Zhengzhou University, No. 1 Jianshe East Road, Zhengzhou, 450052 Henan China; 2 International Institutes of Medicine, The Fourth Affiliated Hospital, Zhejiang University School of Medicine, Yiwu, 322000 Zhejiang China; 3 Department of Pediatrics, Children’s Hospital Affiliated of Zhengzhou University, Zhengzhou, 450052 Henan China; 4 Department of Thoracic Surgery, The First Affiliated Hospital of Zhengzhou University, Zhengzhou University, Zhengzhou, 450052 Henan China; 5 Department of Anesthesiology, The Affiliated Hospital of Southwest Medical University, Luzhou, 646000 Sichuan China; 6 National Health Commission Key Laboratory of Cardiovascular Regenerative Medicine, Heart Center of Henan Provincial People’s Hospital, Central China Fuwai Hospital of Zhengzhou University, Zhengzhou, 451464 Henan China

**Keywords:** Cancer, Molecular biology

## Abstract

Exopolysaccharide (EPS) from *Weissella cibaria* has been devoted to the study of food industry. However, the anticancer activity of *W. cibaria* derived EPS has not yet been investigated. In this study, we obtained the EPS from *W. cibaria* D-2 isolated from the feces of healthy infants and found that D-2-EPS, a homopolysaccharide with porous web like structure, could effectively inhibit the proliferation, migration, invasion and induce cell cycle arrest in G0/G1 phase of colorectal cancer (CRC) cells. In HT-29 tumor xenografts, D-2-EPS significantly retarded tumor growth without obvious cytotoxicity to normal organs. Furthermore, we revealed that D-2-EPS promoted the apoptosis of CRC cells by increasing the levels of Fas, FasL and activating Caspase-8/Caspase-3, indicating that D-2-EPS might induce apoptosis through the extrinsic Fas/FasL pathway. Taken together, the D-2-EPS has the potential to be developed as a nutraceutical or drug to prevent and treat colorectal cancer.

## Introduction

Colorectal cancer (CRC) is the third most common malignancy worldwide and the second leading cause of cancer-related death, with an estimated 1.8 million new cases and approximately 915,880 deaths globally in 2020^1^. At present, treatment mainly includes CRC surgery, radiotherapy, chemotherapy, targeted therapy, immunotherapy, and other comprehensive treatment strategies^2^. Although immunotherapy for the treatment of malignant tumors has brought a new dawn, the overall effect of immunotherapy is not very satisfactory and comes with severe side effects such as drug resistance, organ-specific inflammatory responses, and immune-related adverse events^3^. Therefore, finding new and more efficient CRC prevention and treatment strategies with fewer side effects has become a major scientific problem that urgently needs to be solved in clinical practice.

Previous studies have found that intestinal microorganisms and their components or metabolites are closely related to the occurrence and development of colorectal cancer^4^. Some bacteria in the gut microbiota of CRC patients have been reported to inhibit the occurrence and development of CRC by secreting metabolites such as indole-3-lactic acid (ILA)^5^, reuterin^6^, β-galactosidase^7^ and exopolysaccharide (EPS)^8^. Lactic acid bacteria (LAB), including the well-known lactobacillus, are important members of the intestinal microflora, conferring health benefits such as anti-allergy, antioxidation, immunomodulatory and antitumor effects^9,10^. EPS, as a natural polysaccharide (biopolymer) secreted by LAB into the extracellular environment, is generally recognized as safe (GRAS) and has been found to have numerous health-promoting biological activities, including anticancer activities^11,12^. LAB-derived EPSs are potential anticancer agents through cell apoptosis, anti-proliferation, and cell cycle arrest by regulating tumor-related signaling pathways^12–14^. Additionally, EPS produced by LAB has high biodegradability, nontoxicity, good biocompatibility, and other natural advantages^11^. Therefore, the role of EPSs produced by LAB in tumor prevention has recently received increasing attention.

*Weissella cibaria*, belonging to LAB, was first classified in 2002. *W. cibaria* is widely distributed in traditional fermented foods and the gut^15,16^. Although *W. cibaria* is a relatively new genus compared to other LAB, it has been devoted to the study of the food industry and probiotic potential. Some *W. cibaria* strains have shown considerable potential for health benefits, such as anticancer^17^, antibacterial activities^18,19^, anti-inflammatory activities^20^, enhancement of natural killer cell^21,22^ and protection of the intestinal barrier^23^. Additionally, it has been reported that *W. cibaria* is one of the most important EPS producers of *Weissella* species^24^. EPS from *W. cibaria* exits the characteristics of heat tolerance, antioxidant capacity, and non-digestible by the enzymes in the gastrointestinal. However, most studies of EPS from *W. cibaria* are mainly focused on food industries. There is little information on EPS produced by *W. cibaria* about the health benefits on the host. Furthermore, whether *W. cibaria*-derived EPS can protect humans against CRC, is still unclear.

In this study, we found that the EPS from *W. cibaria* D-2 (D-2-EPS) showed anti-colonic cancer growth effects without obvious toxicity to normal cells in vitro and in vivo. D-2-EPS might suppress the proliferation and viability of CRC cells by activating the Fas/FasL-Caspase8-Caspase3 pathway to induce apoptosis. In addition, D-2-EPS inhibited migration and invasion and resulted in cell cycle arrest of CRC cells in the G0/G1 phase. In light of these findings, D-2-EPS warrants further intensive investigation as a potential nutritional agent or drug for colorectal cancer prevention.

## Methods and materials

### EPS production and isolation

All experiments were performed in compliance with national and institutional laws and are reported in accordance with the ARRIVE guidelines. The *W. cibaria* strain used in this study was isolated from the feces of healthy infants and stored in our lab. The strain was activated using MRS agar plates. Activated *W. cibaria* strains were inoculated at 2% (v/v) inoculum into a modified MRS (mMRS) agar plate with sucrose (10%, m/v) at 30 °C for 48 h in a constant temperature incubator. The bacterial culture was first heated in boiling water for 15 min to inactivate any protein and bacteria and then centrifuged at 12,000×*g* for 30 min at 4 °C to remove bacterial cells and coagulated proteins. Trichloroacetic acid (TCA) was added to the supernatant to achieve a final concentration of 4% (w/v), and the mixture was aged at 4 °C for 12 h. Precipitated proteins were removed by centrifugation (12,000×*g*, 30 min, 4 °C). The TCA step was repeated 3 times until the protein is completely removed. EPSs in the supernatant were precipitated by slowly adding 3 volumes of prechilled anhydrous ethanol (4 °C) and stored at 4 °C for 24 h. Crude EPS was collected by centrifugation (12,000×*g*, 30 min, 4 °C). Then, the crude EPS was dissolved in deionized water, dialyzed (10 kDa, Sangon Biotech, Shanghai, China) against deionized water at 4 °C for 72 h, and then lyophilized^25^. The crude EPS content was analyzed by the phenol‒sulfuric acid method and the sulfuric acid-carbazole method as described by Chen^26^ and Liu^27^, respectively.

### Scanning electron microscopy (SEM) analysis

The D-2-EPS was fixed onto SEM (Zeiss, Jena, Germany) stubs and covered with a thin (10 nm) layer of Au before SEM observation. The surface micromorphology of the EPS sample was observed using SEM with an acceleration voltage of 3.0 kV under 100 × and 1000 × magnification.

### UV spectroscopy analysis

The EPS was dissolved in distilled water and scanned on a spectrophotometer from 200 to 400 nm to observe the UV absorption peaks of the EPS using a UV spectrophotometer (Agilent Technologies, CA, USA).

### Fourier transform infrared (FT-IR) spectroscopy

The specific functional groups of D-2-EPS were characterized by Fourier transform infrared (FT-IR) spectroscopy (Thermo Fisher Scientific, MA, USA). The D-2-EPS powder was ground with potassium bromide (KBr) powder at a ratio of approximately 1:100, and the mixture was pressed into pellets for FT-IR measurement in the wavelength range of 500–4000 cm^−1^.

### Molecular weight analysis

The molecular weight (Mw) of D-2-EPS was performed using a GPC (Agilent 1260 Infinity II, CA, USA) coupled with a refractive index detector (1260 Infinity II). PEG/PEO standards ranging from 600 to 1,500,000 Da were used to plot the standard calibration curve. Mobile phase used was deionized water at a flow rate of 1 mL/min.

### High-performance liquid chromatography (HPLC)

The monosaccharide components of D-2-EPS were analyzed by HPLC with Shimadzu LC-20AD (Shimadzu, Kyoto, Japan) and Xtimate C18 chromatographic columns (4.6*200 mm*5 µm). Approximately 5 mg D-2-EPS was dissolved in 5 mL of trifluoroacetic acid TFA (2 mol/L) and hydrolyzed with sulfuric acid at 120 °C for 4 h. Methanol and nitrogen were added to dry the TFA. The hydrolysate was then neutralized with 1 N NaOH. The column was eluted with pure water at a flow rate of 1 mL/min and detected at 250 nm wavelength. Monosaccharides of EPS were determined by comparison with the retention time of the standard samples, which was plotted using standard sugars including fucose, rhamnose, arabinose, galactose, glucose, fructose, ribose, xylose, mannose, galacturonic acid, glucuronic acid, mannuronic acid, and guluronic acid.

### Cell lines

Two CRC cell lines SW480 (TCHu172) and HT29 (TCHu103) used in this study were purchased from the Cell Bank of the Chinese Academy of Sciences (Shanghai, China). The human normal colon epithelial cell line NCM460 was presented by the Colorectal Surgery Department of the First Affiliated Hospital of Zhengzhou University. SW480 cells were cultured in Dulbecco’s modified Eagle’s medium (Bioss, Beijing, China). HT29 and NCM460 cells were cultured in RPMI-1640 medium (Bioss, Beijing, China). All cells were cultured in medium supplemented with 10% fetal bovine serum (CLARK, VA, USA) and 1% penicillin/streptomycin (Biosharp, Anhui, China) at 37 °C and 5% CO2. The cells used in this study were not split for more than 25 passages.

### CCK-8 assay

SW480, HT29, and NCM460 cells were seeded in a 96-well microplate (2000 cells/well) and preincubated overnight. Then, the cells were cocultured with different concentrations of D-2-EPS (0.2, 0.4, 0.6, 0.8, 1.0, 1.2 mg/mL) for 48 h and 72 h. Cell viability was determined using a Cell Counting Kit-8 (CCK8) (UElandy, Suzhou, China) according to the manufacturer’s instructions. SW480 and HT29 cells treated with 0.7 mg/mL EPS for 72 h were used in the following experiments.

### Clonal formation assay

1 × 10^3^ treated SW480 and HT29 cells were seeded in 12-well plates. SW480 was cultured with DMEM and HT29 was cultured with RPMI-1640 for 14 days, the single clones were fixed with 4% paraformaldehyde for 20 min and stained with Giemsa (Solarbio, Beijing, China) for 15 min. The 12-well plate was rinsed with running distilled water and then inverted on a white background plate to count the number of clones. Clone formation rate = the number of clones/number of inoculated cells × 100%.

### Wound healing assay

Treated HT29 and SW480 cells were plated into 12-well plates with 5 × 10^5^ cells/well. The scratch experiment was performed until the cells covered the bottom of the well. Aspirate the cell culture medium from wells and wash the cells twice with PBS. And then micropipette tip was used to make a cross in the center. The floating cells were washed off with PBS, and wells were added to serum-free medium. Photographs were taken at 0 and 48 h until the cells migrated to the scratched area or the scratches healed. The migration area (%) was determined by measuring the wound healing percentage (%).

### Transwell assay

For transwell migration, 1 × 10^5^ treated HT29 and SW480 cells were seeded in upper chambers with 8-μm pore membranes of 24-well plates (Corning, NY, USA). RPMI 1640 or DMEM with 20% FBS was added to lower chambers to induce cell migration. Then, the migrated cells were fixed in 4% paraformaldehyde for 20 min and stained with Giemsa (Solarbio, Beijing, China) after 12 h. Images of the migrating cells were captured in five fields using an optical microscope. Similarly, for the transwell invasion assay, 2 × 10^5^ treated HT29 and SW480 cells were seeded in upper chambers coated with Matrigel (Corning, NY, USA). The next steps are the same as transwell migration. The invasive cells were stained and imaged. All data processing was performed by ImageJ.

### Cell apoptosis analysis

HT29 and SW480 cells were inoculated into 6-well plates and treated with D-2-EPS. After 72 h cell culture, floating and adherent cells in each well were harvested and double-stained with Annexin-V-FITC and propidium iodide (PI) in the dark for 15 min at 4 °C. Then, cell apoptosis was assessed by flow cytometry (ACEA, CA, USA) immediately.

### Cell cycle analysis

HT29 and SW480 cells were seeded in 6-well plates and treated with D-2-EPS. After 72 h cell culture, adherent cells in each well were harvested and fixed in 70% ethanol at 4 °C overnight. After washing with PBS and centrifugation at 1000 rpm for 5 min, the cells were subsequently stained with PI in the dark for 30 min at 4 °C. Then the stained cells were detected by flow cytometry (ACEA, CA, USA) within 24 h.

### Animal models

Four-week-old male athymic BALB/c nude mice (SPF (Beijing) Biotechnology Co., Ltd.) were purchased and housed in a specific pathogen-free (SPF) environment under a 12-h light/dark cycle at constant temperature and humidity. HT29 cells (0.1 ml, 5 × 10^6^/mL) were subcutaneously injected into the right axilla of mice to induce xenograft tumors. After the tumor volume reached 200 mm^3^, the mice were injected intratumorally every 4 days as follows: 0.1 mL phosphate-buffered saline (control group), 50 mg/kg EPS (low-dose EPS group), 100 mg/kg EPS (medium-dose EPS group), or 150 mg/kg EPS (high-dose EPS group). The tumor and body weight growth were determined every day. Tumor volumes were calculated using an established formula (½ length × width^2^). After 15 days, the mice were sacrificed, the tumors were removed for IHC, and the heart, liver, spleen, and kidney were harvested for HE staining.

### Western blot (WB) assay

Protein in HT29 cells was extracted with RIPA buffer containing PMSF (Solarbio, Beijing, China). The protein concentration was determined using a BCA protein assay kit (Solarbio, Beijing, China). Loading buffer was added to the protein samples, and the mixture was boiled for 5 min. The mixture containing 30 μg protein was loaded onto 10% SDS‒PAGE gels to separate the proteins before being transferred to a polyvinylidene difluoride (PVDF) membrane. The membrane was incubated with 5% skim milk at room temperature for 2 h and then the blots were cut according to molecular weight before incubating with diluted primary antibody at 4 °C overnight. After being washed three times with Tris-buffered saline with Tween 20 (TBST), the membrane was incubated with the secondary antibody for 1 h at room temperature. The protein bands were measured and quantified. The following antibodies were used: anti-β-actin (CST, #3700); anti-GAPDH (HUABIO, ET1601-4); anti-Fas (Affinity, AF5342); anti-FasL (Affinity, AF0157); anti-caspase8 (Asp384) (Affinity, AF6442); anti-cleaved caspase8 (Affinity, AF5267); anti-caspase3 (CST, #9662); and anti-cleaved caspase3 (Asp175) (CST, #9661).

### Hematoxylin and Eosin (H&E) staining and immunohistochemistry (IHC) assay

The organs (heart, liver, spleen, lung, and kidney) and tumors were collected, dehydrated with graded alcohol, soaked in xylene solution, and then embedded in paraffin. The paraffin tissue sections (5 μm thickness) were dewaxed in xylene, soaked in graded alcohol, and dehydrated. After staining with H&E, the samples were photographed by microscopy (Olympus, Tokyo, Japan).

Deparaffinized and dehydrated paraffin tissue sections (5 μm thickness) were obtained. The cells were treated with 3% hydrogen peroxide at room temperature for 10 min in the dark to block the intrinsic catalase. Then, the sections were subjected to hot antigen retrieval with 10 mM sodium citrate buffer (pH 6.0). Normal serum was used to block nonspecific binding sites at 37 °C for 1 h. Ki67 (Abcam, ab16667) was treated overnight at 4 °C, and the secondary antibody was added at 37 °C for 30 min. Streptavidin-peroxidase complex was added for 30 min. Furthermore, sections were stained and re-stained using DAB and hematoxylin. After washing with running water, the samples were treated with graded alcohol, cleared to xylene, and then mounted for microscopy. IHC images were acquired using a fluorescence microscope system (Olympus, Tokyo, Japan).

### Statistical analysis

Experiments were carried out in triplicate, and 3–4 replicate wells were set in each group. All data were analyzed using GraphPad Prism 9.0 (GraphPad, San Diego, CA, USA) and expressed as the Mean ± SEM. One-way analysis of variance (ANOVA) and T-test were used to determine significant differences (p ≤ 0.05) for different samples. Adobe Illustrator 2020, Adobe Photoshop 2020, and ImageJ software were used for the stitching of pictures.

### Ethics approval and consent to participate

Ethical approval for the involvement of human subjects in this study was granted by the Ethics Committee of The First Affiliated Hospital of Zhengzhou University, Reference number 2022-KY-0231-002, 4/12/2022. All animal experiments were carried out in accordance with the National Research Council's Guide for the Care and Use of Laboratory Animals and approved by the Institutional Animal Care and Use Committee of Zhengzhou University (No. 2021042501).

### Informed consent

Informed consent was obtained from all subjects and/or their legal guardian(s).

## Results

### Purification and characterization of EPS from *W. cibaria* D-2

**Figure 1:**
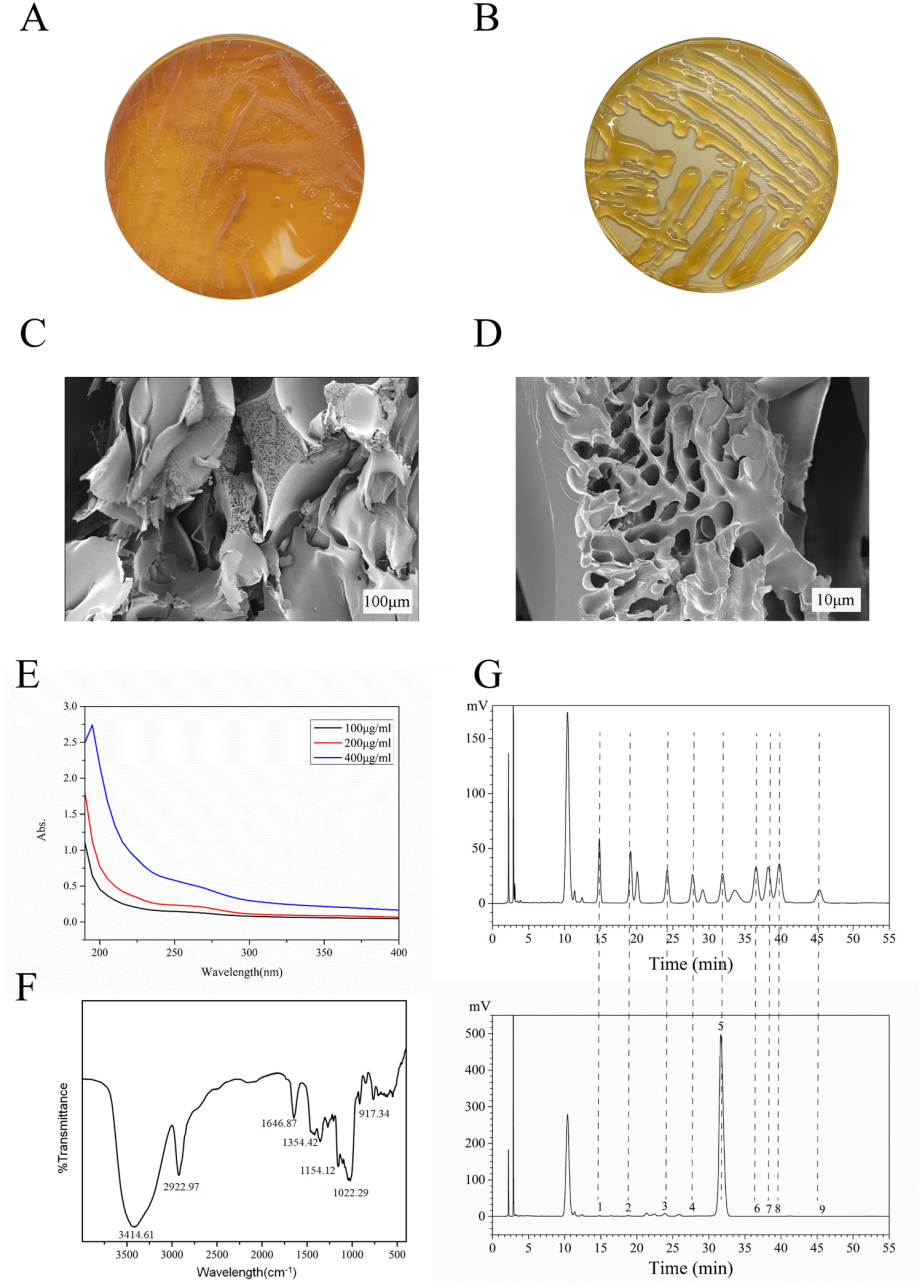
Characterization of crude D-2-EPS. (**A**,**B**) The characteristics of *W. cibaria* D-2 grown on MRS plate (**A**) and mMRS plate (**B**). (**C**,**D**) SEM images of D-2-EPS at × 100 (**C**) and × 1000 (**D**) magnification. (**E**) Ultraviolet (UV)-visible spectrum of D-2-EPS. (**F**) FT-IR spectra of D-2-EPS. (**G**) High-performance liquid chromatography (HPLC) chromatograms of standard monosaccharides and D-2-EPS. Peaks: 1. mannose; 2. ribose; 3. glucuronic acid; 4. galacturonic acid; 5. glucose; 6. galactose;7. xylose; 8. arabinose; 9. fucose.

Sucrose was reported to be a great carbon source for EPS production by *W. cibaria*^28^. We cultured the D-2 strain, which was isolated from the feces of healthy infants in MRS and mMRS (supplemented with 100 g/L sucrose) agar plates, respectively. Then, the colony phenotypes in different media were observed. The results showed that *W. cibaria* colonies grown on mMRS plates exhibited obvious mucoid and slimy characteristics compared with those colonies grown on MRS plates (Fig. 1A,B), proving that EPS production was promoted by sucrose. The crude EPS of *W. cibaria* D-2 was then extracted by previously reported methods^25,29^, and the EPS yield was 39.106 g/L after 48 h of culture. Most of the EPS yields of *W. cibaria* strains reported in previous literature ranged from 1.12 to 10.8 g/L^30,31^, except for the EPS from *W. cibaria* JAG8 reached 40 g/L^32^, indicating that strain D-2 may have great potential to produce more EPS by optimizing fermentation parameters. These results indicated that *W. cibaria* D-2 used in this study could produce high contents of EPS and were suitable for further study.

SEM imaging is beneficial for observing the micromorphology of biomacromolecules and understanding their physical and functional characteristics. The lyophilized D-2-EPS presented a fluffy and white flocculation shape. D-2-EPS on analysis by SEM were appeared as porous web like structure (Fig. 1C,D). The porous structure confers high water-holding capacity to EPS, which makes it a potential additive for various food products as reported by Purama et al.^33^. EPS can be a homopolysaccharide formed by the polymerization of a single type of monosaccharide molecule or a heterosaccharide formed by the polymerization of different types of monosaccharide molecules or their derivatives. The common saccharide derivative of EPS is uronic acid^25^. The total saccharides in D-2-EPS accounted for 85.15% (w/w) by the phenol‒sulfuric acid method, while the saccharide derivatives accounted for 9.12% (w/w) by the sulfuric acid-carbazole method. D-2-EPS were scanned by a Shimadzu ultraviolet (UV)-visible spectrophotometer. As shown in Fig. 1E, there was no obvious absorption observed at 280 nm, demonstrating that D-2-EPS fractions lacked protein. These results indicated that we obtained a high-purity polysaccharide sample.

**Figure 2:**
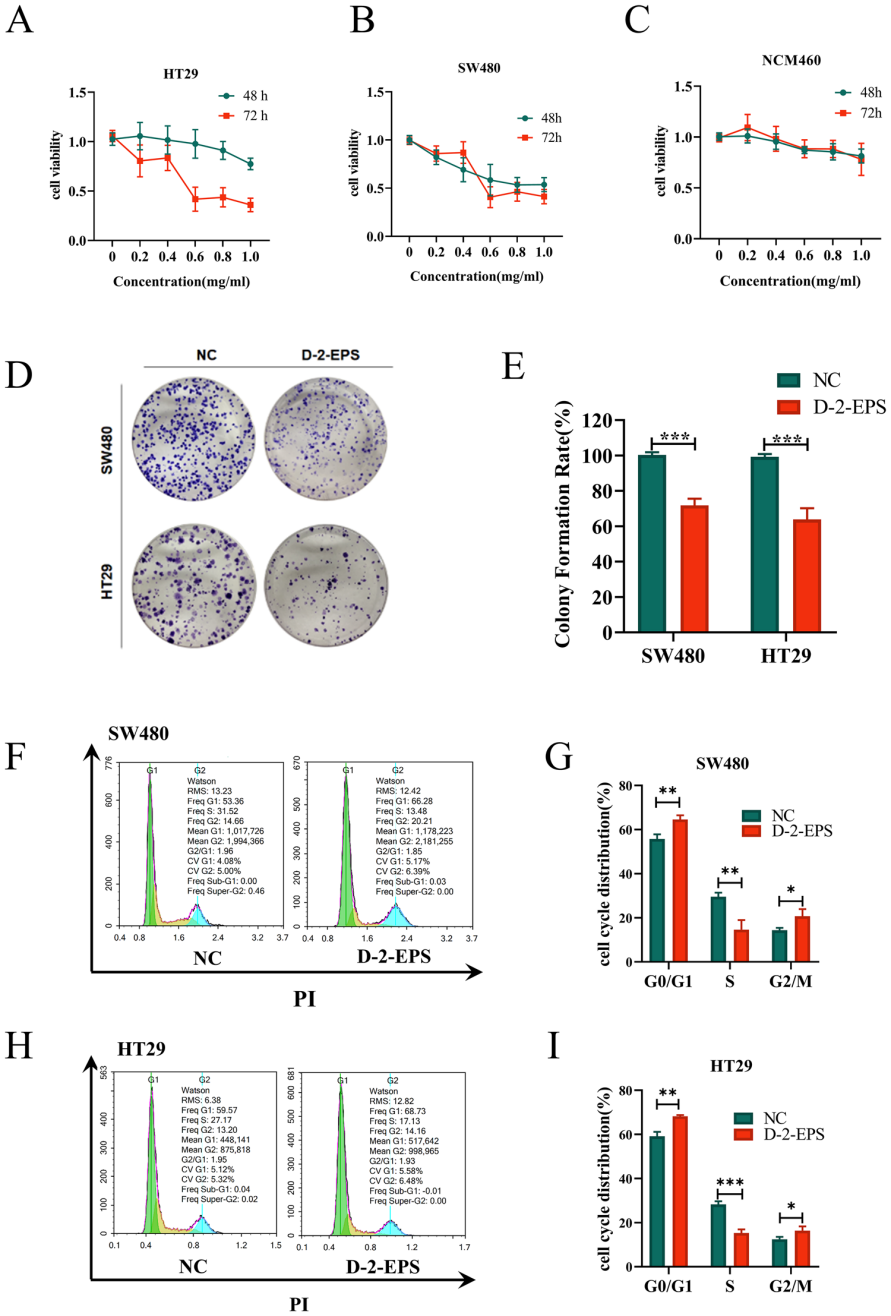
D-2-EPS inhibited the proliferation and induced cell cycle arrest of CRC cells. (**A**–**C**) CCK8 assay of HT29 (**A**), SW480 (**B**), and NCM460 (**C**) cultured with D-2-EPS after 48 h, 72 h. (**D**) Clonal formation assay of SW480 and HT29 cultured with D-2-EPS after 72 h. (**E**) Quantitative analysis of clone number of SW480 and HT29. (**F**) The cell cycle distribution of SW480 treated with D-2-EPS. (**G**) Quantitative analysis of the proportion of SW480 in different cell cycle phases. (**H**) The cell cycle distribution of HT29 treated with D-2-EPS. (**I**) Quantitative analysis of the proportion of HT29 in different cell cycle phases. Values represent Mean ± SEM of the mean. Student's t-test, *P-value < 0.05, **P-value < 0.01, ***P-value < 0.001, ****P-value < 0.0001.

Different linkages and groups in polysaccharides can absorb different infrared wavelengths. The functional groups and bond configuration of D-2-EPS were detected by FT-IR spectroscopy. As shown in Fig. 1F, D-2-EPS fractions possessed typical absorption bands of polysaccharides. The absorption peak at 3414.61 cm^−1^ was attributed to the stretching vibration of –OH in the polysaccharide molecule, and the sharp peaks at 2922.97 cm^−1^ and 1354.42 cm^−1^ were attributed to the stretching vibration and the variable angle vibration of C–H in the methyl or methylene group of the polysaccharide molecule^34^. The stretching vibration of C=O at 1646.87 cm^−1^ proved that the sample contained uronic acid^35^. Uronic acids are usually present in the form of pyranose and furanose^36^. The absorption peaks at 1154.12 cm^−1^ and 1022.29 cm^−1^ represented the C–O–H bending vibration and the C–O–C stretching vibration, respectively^37^, demonstrating the presence of a pyranose ring. The peak at 912 cm^−1^ was caused by the asymmetric stretching vibration of the pyran ring^38^, proving the existence of uronic acid.

Molecular weight and monosaccharide composition of EPS are the two key features which determine the properties of the EPS. From GPC analysis, it was calculated that D-2-EPS has a molecular weight of 1160 kDa. The composition of monosaccharide and uronic acid in D-2-EPS was analyzed by high-performance liquid chromatography (HPLC). As shown in Fig. 1G and Table 1, D-2-EPS was mainly composed of the majority of glucose (98.426% area). Collectively, D-2-EPS was considered to be a homopolysaccharide.

**Table 1:** The composition of D-2-EPS.

Monosaccharide	Retention time (min)	Height%
Mannose	14.854	0.169
Ribose	18.827	0.178
Glucuronic acid	23.894	1.103
Galacturonic acid	27.441	0.039
Glucose	31.662	98.426
Galactose	36.646	0.021
Xylose	38.364	0.032
Arabinose	39.940	0.022
Fucose	46.010	0.009

### D-2-EPS inhibited the proliferation of CRC cells

Tumor cells have a stronger proliferation ability than normal cells. To fully understand the effect of the anticancer ability of D-2-EPS, CCK8 assays were performed to detect the proliferation ability of CRC cells. The results showed that D-2-EPS suppressed the growth of CRC cells (Fig. 2A,B). Effective antitumor agents need to selectively kill cancer cells with minimal cytotoxicity toward normal cells. Thus, the cytotoxicity of D-2-EPS on normal epithelial cell line NCM460 was also measured. The results showed that D-2-EPS had no obvious toxicity on NCM460 cells even at high concentrations (Fig. 2C). The IC50 of SW480, HT29 and NCM460 treated with D-2-EPS for 72 h were about 0.52 mg/mL, 0.58 mg/mL and 1.2 mg/mL, respectively. Therefore, the CRC cells treated with 0.7 mg/mL D-2-EPS for 72 h were used in the following experiments. This condition of action inhibited CRC cells up to 50%, while being essentially non-toxic to normal intestinal epithelial cell NCM460.

Next, we used the clonal formation assay to further verify the long-term inhibitory effect of D-2-EPS on CRC cell proliferation at the single-cell level. Consistent with the results of the CCk8 assay, D-2-EPS treatment impaired the clone formation ability of SW480 and HT29 (Fig. 2D,E), further suggesting that D-2-EPS could inhibit the proliferation of CRC cells.

### D-2-EPS induced cell cycle arrest of CRC cells

Cell cycle regulation is vital for cell proliferation, growth, and repair^39^. Thus, we further determined the effect of D-2-EPS on the cell cycle of CRC cells. The results of flow cytometry revealed that the proportion of CRC cells in G0/G1 and G2 phases significantly increased after D-2-EPS treatment, while cells in S phase decreased correspondingly (Fig. 2F–I). These results suggested that D-2-EPS possessed anti-proliferation activity on CRC cells by inducing cell cycle arrest in G0/G1 phase.

The normal cell cycle can be divided into the intermittent phase and mitotic phase (M), and interphase is often divided into the resting phase (G0), pre-DNA synthesis phase (G1), DNA synthesis phase (S), and DNA synthesis anaphase (G2)^40^. Among them, the G1/S transition is an important checkpoint and is responsible for the initiation and completion of DNA replication. The whole cycle can be expressed as G0 → G1 → S → G2 → M. If the division process of tumor cells is blocked, the division stays in a certain stage of interphase, and mitosis cannot proceed smoothly. Thus, the proliferation and spread of tumor cells can be effectively inhibited^40^. It has been reported that many EPSs produced by LAB, in addition to *W. cibaria,* can induce cell cycle arrest in G0/G1 phase of CRC cells^12^.

### D-2-EPS inhibited the migration and invasion of CRC cells

**Figure 3:**
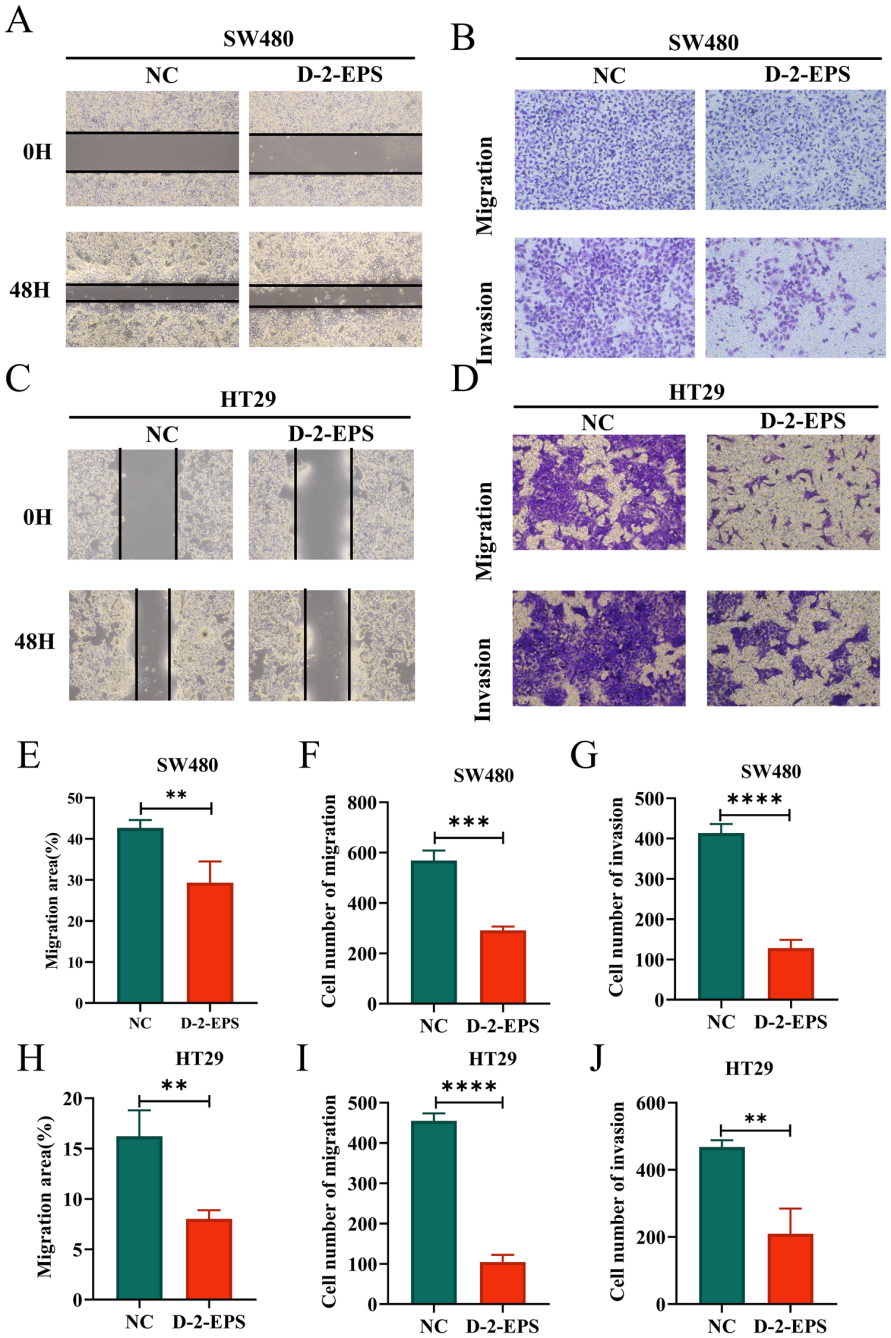
D-2-EPS inhibited the migration and invasion of CRC cells. (**A**) The wound closure of SW480. (**B**) The assays transwell migration and transwell invasion of SW480. (**C**) The wound closure of HT29. (**D**) The assays transwell migration and transwell invasion of HT29. (**E**) Quantitative analysis of migration area of SW480. (**F**) Quantitative analysis of migration cell number of SW480. (**G**) Quantitative analysis of invasion cell number of SW480. (**H**) Quantitative analysis of migration area of HT29. (**I**) Quantitative analysis of migration cell number of HT29. (**J**) Quantitative analysis of invasion cell number of HT29. Values represent Mean ± SEM of the mean. Student's t-test, *P-value < 0.05, **P-value < 0.01, ***P-value < 0.001, ****P-value < 0.0001.

Migration and invasion are important factors in the motility of tumor cells to infiltrate and metastasize^41^, which was detected by wound healing assays and transwell assays in this study. Wound healing assay results showed that D-2-EPS treatment slowed the wound closure of CRC cells, indicating that the migration ability of the CRC cells was weakened by D-2-EPS (Fig. 3A,C,E,H). Furthermore, the results of transwell migration assays revealed that fewer CRC cells migrated to the lower chamber in the D-2-EPS group, indicating a weak migration ability of cells treated with D-2-EPS, which was consistent with the results of wound healing assay (Fig. 3B,D,F,I). Additionally, the results of the transwell invasion assays illustrated that the number of cells that penetrated the matrigel to the bottom chamber in D-2-EPS group was significantly less than that of the control group (Fig. 3B,D,G,J), reflecting that D-2-EPS significantly inhibited CRC cells invasion. Taken together, the D-2-EPS possessed anti-migration and anti-invasion abilities against CRC cells.

### D-2-EPS induced apoptosis in CRC cells

**Figure 4:**
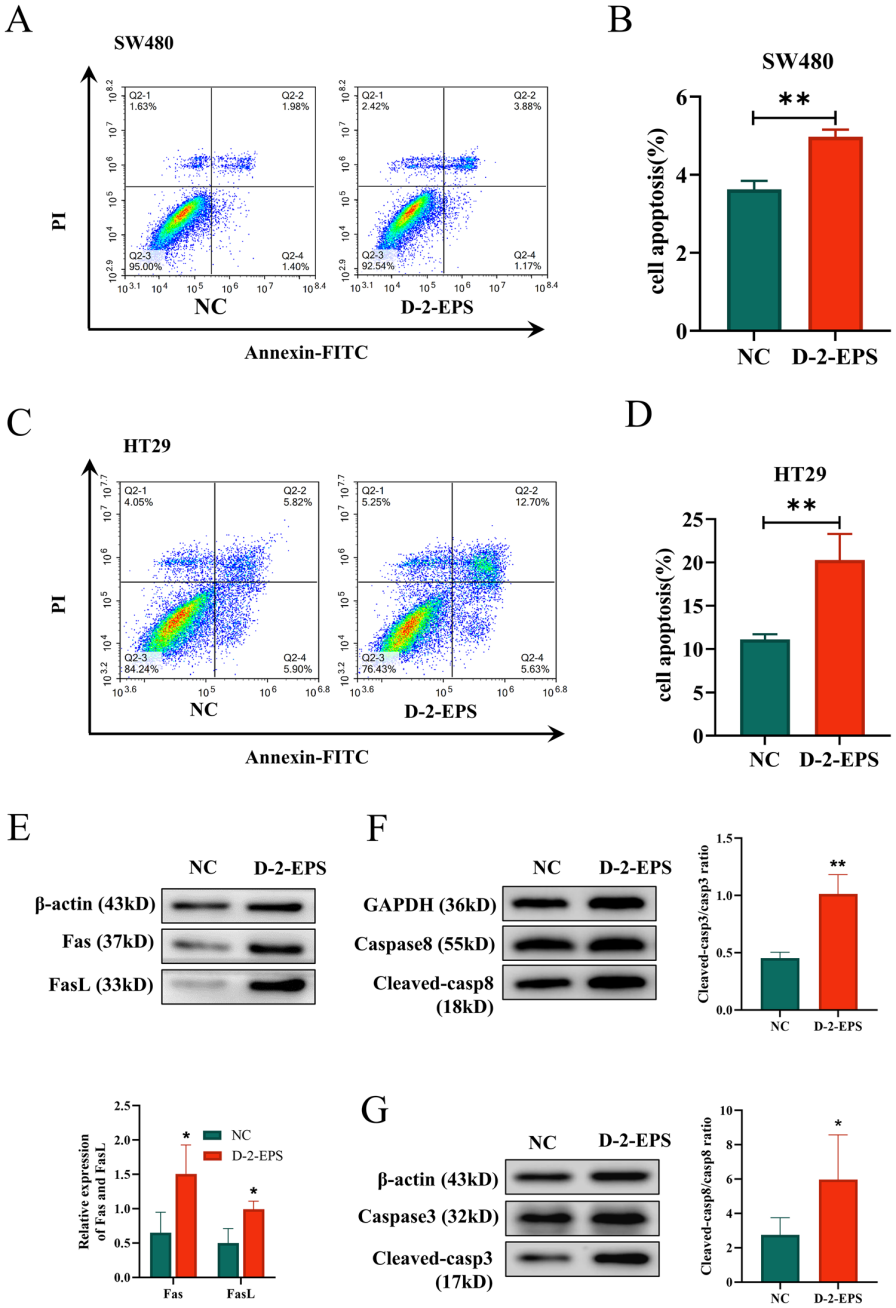
D-2-EPS induced cell apoptosis of CRC cells. (**A**) The cell apoptosis of SW480 treated with D-2-EPS. (**B**) Quantitative analysis of apoptosis of SW480. (**C**) The cell apoptosis of HT29 treated with D-2-EPS. (**D**) Quantitative analysis of apoptosis of HT29. (**E**) The protein levels of Fas, FasL in HT29. (F) The protein levels of caspases-8 and cleaved caspases-8 in HT29. (**G**) The protein levels of caspase-3 and cleaved caspase-3 in HT29. The blots were cut prior to hybridisation with antibodies. Values represent Mean ± SEM of the mean. Student's t-test, *P-value < 0.05, **P-value < 0.01, ***P-value < 0.001, ****P-value < 0.0001.

To explore whether the suppressive effects of D-2-EPS on the proliferation of SW480 and HT29 cells were mediated by apoptotic induction, we labeled the early and late apoptotic cells with Annexin-V and PI, respectively. The total proportion of apoptotic cells was equal to the sum of the proportion of early and late apoptotic cells. As shown in Fig. 4A–D, D-2-EPS treatment increased the proportion of apoptotic CRC cells, indicating D-2-EPS significantly promoted apoptosis of HT29 and SW480 cells.

We next determined the expression of apoptosis-related proteins in HT29 cells to further investigate the underlying mechanism for cell apoptosis induced by D-2-EPS. We found that D-2-EPS treatment group could up-regulate the co-expression of tumor necrosis factor receptors (Fas) and its ligand (FasL) compared to the control group (Fig. 4E). Likewise, the expression of cleaved caspases-8 protein was increased by D-2-EPS treatment, followed by the activation of downstream caspase-3 protein, and a subsequent increment of cleaved caspase-3 expression, which eventually caused the apoptosis of CRC cells (Fig. 4F,G). All in all, the expression of proteins associated with the death receptor pathway proved that D-2-EPS triggered HT29 cell apoptosis by the Fas/FasL mediated caspase-dependent death pathway.

### D-2-EPS inhibited tumor growth in vivo

**Figure 5:**
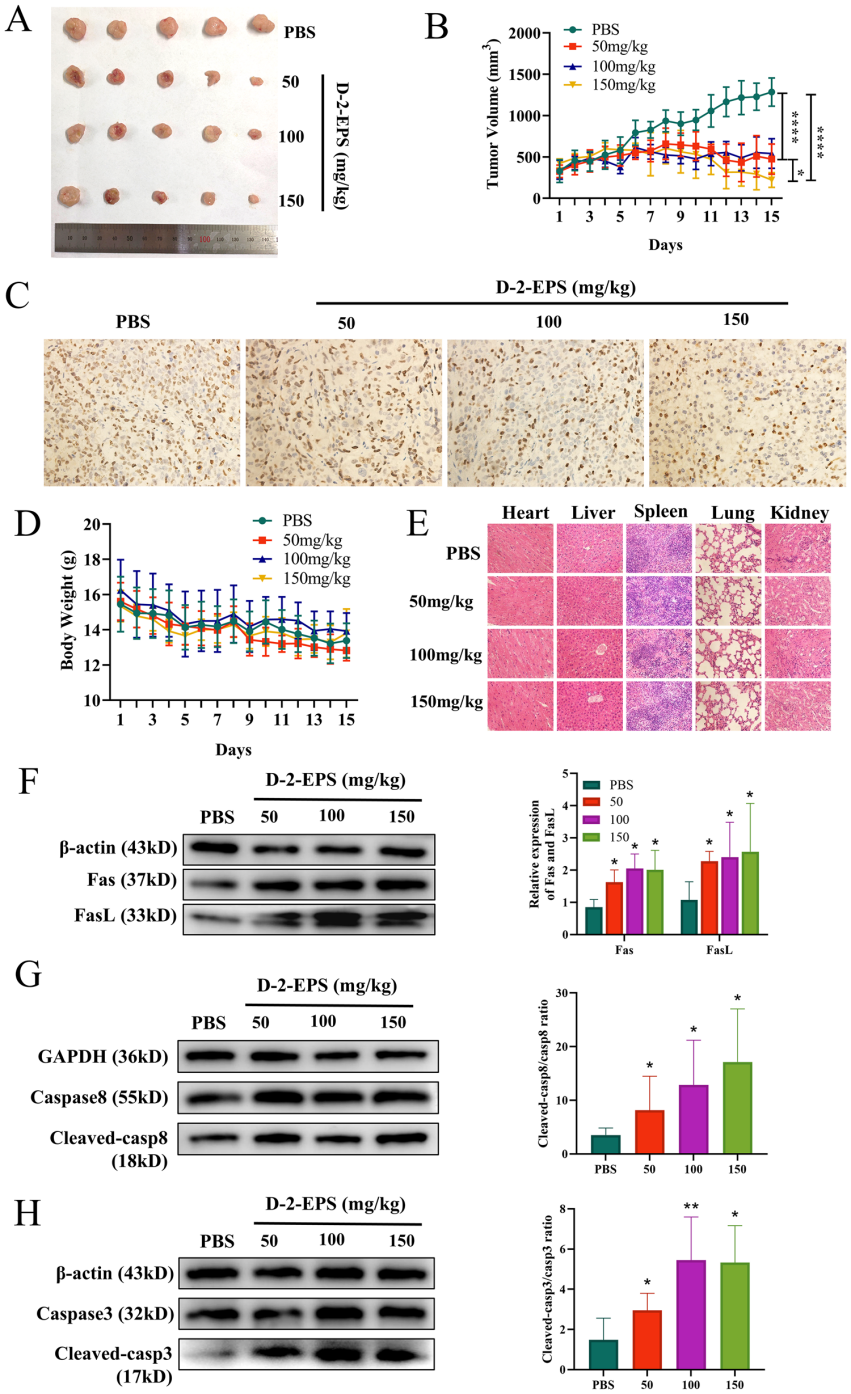
D-2-EPS inhibited tumor growth in vivo. The images of tumor (**A**), the growth curves of tumor volume (**B**), the immune-histochemical result of Ki67 of the tumor tissue (**C**), and the body weight (**D**) in the HT29 tumor xenograft model. (**E**) The HE-stained images of the heart, liver, spleen, lung, and kidney. (**F**) The protein levels of Fas, FasL. (**G**) The protein levels of caspases-8 and cleaved caspases-8. (**H**) The protein levels of caspase-3 and cleaved caspase-3. The blots were cut prior to hybridisation with antibodies. Values represent Mean ± SEM of the mean. Student's t-test, *P-value < 0.05, **P-value < 0.01, ***P-value < 0.001, ****P-value < 0.0001.

The inhibitory function of D-2-EPS on the growth of subcutaneous HT29 xenografts in nude mice was further evaluated. After the successful construction of the HT29 subcutaneous tumor-bearing model, nude mice were randomly divided into 4 groups, which were treated with PBS, 50 mg/kg D-2-EPS, 100 mg/kg D-2-EPS, and 150 mg/kg D-2-EPS by intratumoral injection. The in vivo results showed that administration of D-2-EPS significantly suppressed the tumor growth of HT29 tumor-bearing mice compared to that of PBS treatment (Fig. 5A,B), and the inhibitory effect of 150 mg/kg D-2-EPS was better than that of the 50 mg/kg and 100 mg/kg D-2-EPS.

Ki67 is a proliferation marker reflecting the proliferative ability of tumor cells^42^. To further confirm the inhibitory effect of D-2-EPS on the proliferation of tumor cells, the expression of Ki67 was verified in HT-29 tumor-bearing mice by IHC staining. The results showed that the expression of Ki67 in the three groups treated with different concentrations of D-2-EPS was significantly lower than that in the PBS group, which was consistent with the differential trend of tumor growth (Fig. 5C). Furthermore, the toxicity of D-2-EPS to normal mouse organs was further analyzed. As shown in Fig. 5D, D-2-EPS had no obvious effect on mouse body weight. Meanwhile, The HE-stained images of the normal organs, such as the heart, liver, spleen, lung, and kidney, were similar to those of mice in the control group (Fig. 5E). These results indicated that D-2-EPS could inhibit tumor growth in mice without obvious toxicity or side effects on body weight and important organs. The levels of proteins associated with the death receptor-mediated apoptosis signaling pathway (Fas/FasL mediated caspase-dependent death pathway) were also detected in HT29 xenografts and the results were consistent with those in HT29 cells (Fig. 5F–H).

## Discussion

Due to the absence of virulence factors and antibiotic-resistance genes, most *W. cibaria* strains are considered to be safe^24^. The application of *W. cibaria* in the probiotic field has drawn the attention of researchers in recent years. *W. cibaria* CMU, which has been used as commercial probiotics in Korea, is known to improve periodontitis by inhibiting the activation of NF-κB signal induced by periodontal pathogens^20^. Huang et al. evaluated the protective effect of *W. cibaria* MW01 on the intestinal epithelial barrier challenged by LPS. They found that strain MW01 remarkably attenuated the inflammatory response and regulated the levels of tight junction proteins^23^. *W. cibaria* JW15 was found to induce the production of cytokines (TNF-α, IL-6, IL-1β) and immunoglobulin, enhancing the activity of natural killer cells^21,22^. It has been reported that synbiotic soymilk and xylooligosaccharides complex fermented by *W. cibaria* FB069 can inhibit the proliferation of Caco-2 and HCT116 cells by reducing the transcriptional levels of MD2, TLR4, MyD88, and NF-κb^43^.

EPS are important secondary metabolites of *W. cibaria*, which has been widely applied in the food processing as a thickener and stabilizer^44,45^. Recently, researchers have realized that further explorations are worthwhile for tapping the therapeutic potential of EPSs produced by *W. cibaria*. Baruah et al. found that the EPS from *W. cibaria* RBA12, a strain isolated from pomelo, was beneficial for the growth of *Bifidobacterium* and *Lactobacillus *spp.^46^. The fructan EPS derived from *W. cibaria* MD2 not only increases the tolerance of *C. elegans* against oxidative stress, but also extended *C. elegans* lifespan through the activation of DAF-16 nuclear localization^47^. Kibar et al. revealed that EPS produced by *W. cibaria* EIR/P2 is glucan with beneficial properties including antimicrobial and antioxidant activity, inhibition of biofilms, and promotion of the viability of periodontal ligament fibroblasts^48^. However, the beneficial effect of *W. cibaria* producing EPS on human health, including the field of anti-tumor treatment, was still rare. In this study, we obtained a homopolysaccharide D-2-EPS from *W. cibaria* D-2 isolated the feces of healthy infants, and found D-2-EPS could inhibit proliferation, migration, and invasion of CRC cells in vitro, and suppressed the tumor growth in HT-29 tumor xenografts. To the best of our knowledge, this study represents the first conducted for the suppressive effect of *W. cibaria* derived EPS on CRC cells.

EPS has shown great promise for pharmaceutical applications, however, limited EPS production of lactobacillus with beneficial effects restricts their large-scale industrial application. Optimization of the fermentation parameters, such as carbon sources, nitrogen sources, inorganic salts, fermentation time, and incubation temperature, play an important role in enhancing the production of EPS^30^. Additionally, the role of physiological and genetic regulation in the enhancement of EPS yield has long been appreciated. With increasing research on biosynthesis pathways, as well as the rapid development of molecular biology technologies, metabolic regulation of EPS biosynthesis to change the production and structure through genetic engineering methods has attracted more and more attention. However, it has been a long-pursued goal in isolation of wild type lactobacillus strains with high yield of EPS. In this study, 10% sucrose was selected as the carbon source for the fermentation of *W. cibaria* D-2 to produce EPS. Surprisingly, The EPS production was up to 40 g/L without an optimization process. The high EPS yield provided important reassurance for future clinical translation. Next, we will further optimize the fermentation conditions to fully exploit the potentialities of *W. cibaria* D-2 to produce EPS. Additionally, genomes sequencing will also be performed to elucidate the mechanism of EPS production by *W. cibaria* D-2 from genetic levels.

EPS suppresses tumor development or growth through a variety of mechanisms, including anti-proliferation, apoptosis induction, cell cycle arrest, anti-mutagenesis, anti-angiogenesis, and anti-inflammatory action^12^. It has been well known that resistance to apoptosis signals is one of the striking hallmarks of cancer cells^49^. Thus, it is an efficient and common scheme to make tumor cells in response to apoptosis signals in antitumor research. Meanwhile, various studies reported that EPSs from LAB have shown protective effects against CRC by promoting apoptosis. EPSs isolated from LAB promote cell apoptosis of CRC cells via two main pathways involving either the death receptor pathway (extrinsic pathway) or the mitochondrial pathway (the intrinsic pathway). Of note, there is only one study reported that EPS, obtained from *L. plantarum* NCU116, can induce apoptosis of CT26 cells by activating FAS and FASL (extrinsic pathway)^50^. Meanwhile, the intrinsic mitochondrial pathway seems to be more common in the apoptotic CRC cells treated with LAB EPSs^13,25,51^. In this study, we showed for the first time that *W. cibaria* EPS promotes the apoptosis of CRC cells through the extrinsic FAS/FASL pathway. Although more in-depth and specific apoptosis-related investigations need to be performed for further inference of the possible signaling pathways and mechanisms, our study holds considerable promise for D-2-EPS as a possible therapeutic agent for CRC. EPS combined with other antitumor agents has shown excellent synergistic treatment effects in inhibiting tumor growth. While, whether D-2-EPS has synergistic effects on tumor therapy with other treatments still needs further studies.

## Conclusion

In conclusion, D-2-EPS showed anti-colonic cancer growth effects without obvious toxicity to normal cells in vitro and in vivo. D-2-EPS might suppress the proliferation and viability of CRC cells by activating the Fas/FasL-Caspase8-Caspase3 pathway to induce apoptosis. In addition, D-2-EPS inhibited migration and invasion and resulted in cell cycle arrest of CRC cells in G0/G1 phase. In light of these findings, D-2-EPS warrants further intensive investigation as a potential nutritional agent or drug for colorectal cancer prevention (Supplementary Information S1).

### Supplementary Information

Supplementary Information.

## Data Availability

The datasets used and/or analysed during the current study available from the corresponding author on reasonable request.

## Abbreviations

EPS: Exopolysaccharide
CRC: Colorectal cancer
ILA: Indole-3-lactic acid
LAB: Lactic acid bacteria
GRAS: Generally recognized as safe
mMRS: Modified MRS
SEM: Scanning electron microscopy
FT-IR: Fourier transform infrared
HPLC: High-performance liquid chromatography
