# Supplementary material for: The anticancer mechanisms of exopolysaccharide from Weissella cibaria D-2 on colorectal cancer via apoptosis induction
Source: Sci Rep. 2023 Nov 30;13:21117. doi: 10.1038/s41598-023-47943-7 (PMC10689803; doi:10.1038/s41598-023-47943-7)

Original western blots images for Figure 4E

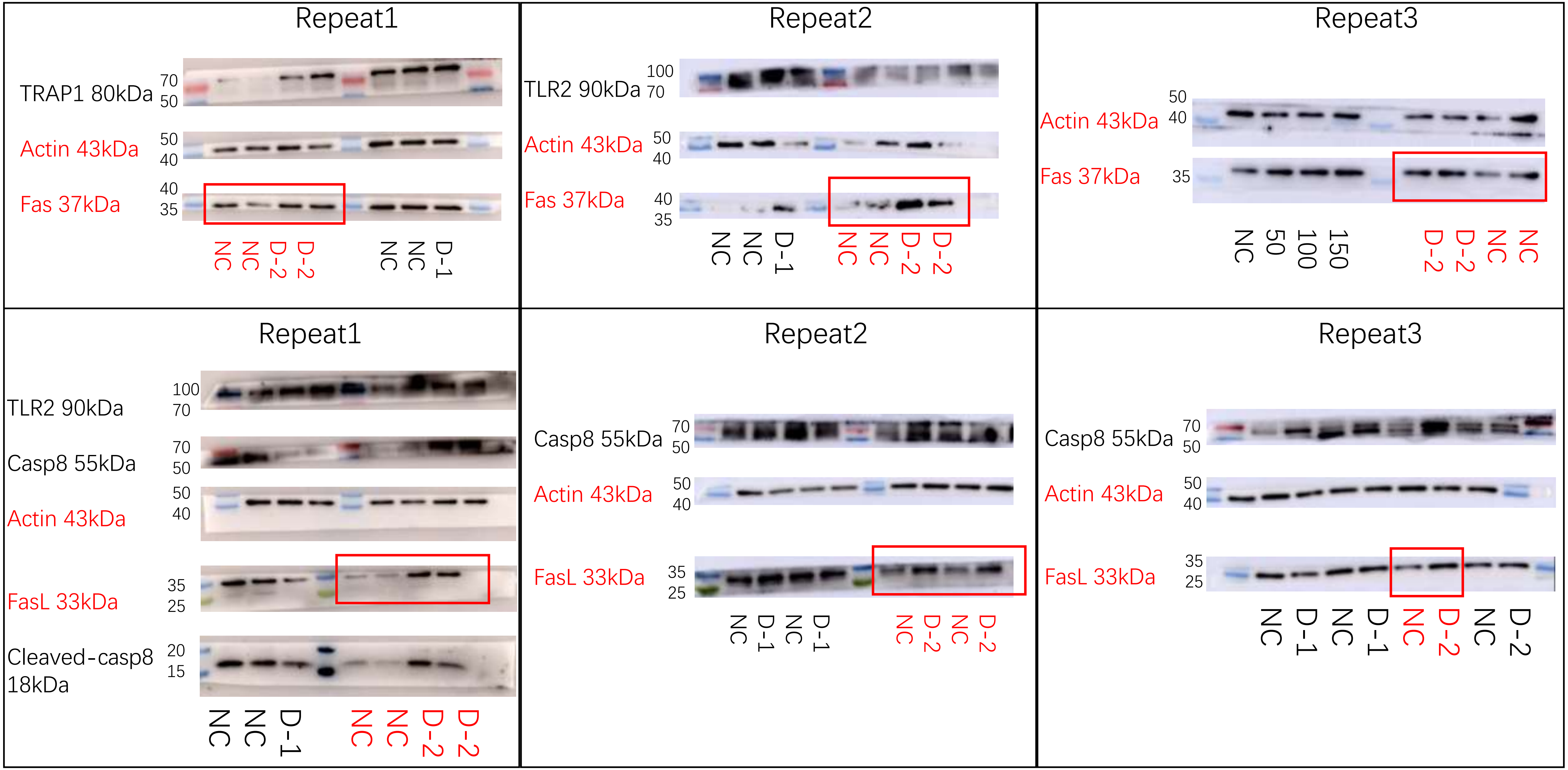

All blots in this file were cut prior to hybridisation with antibodies during blotting.

Original western blots images for Figure 4F

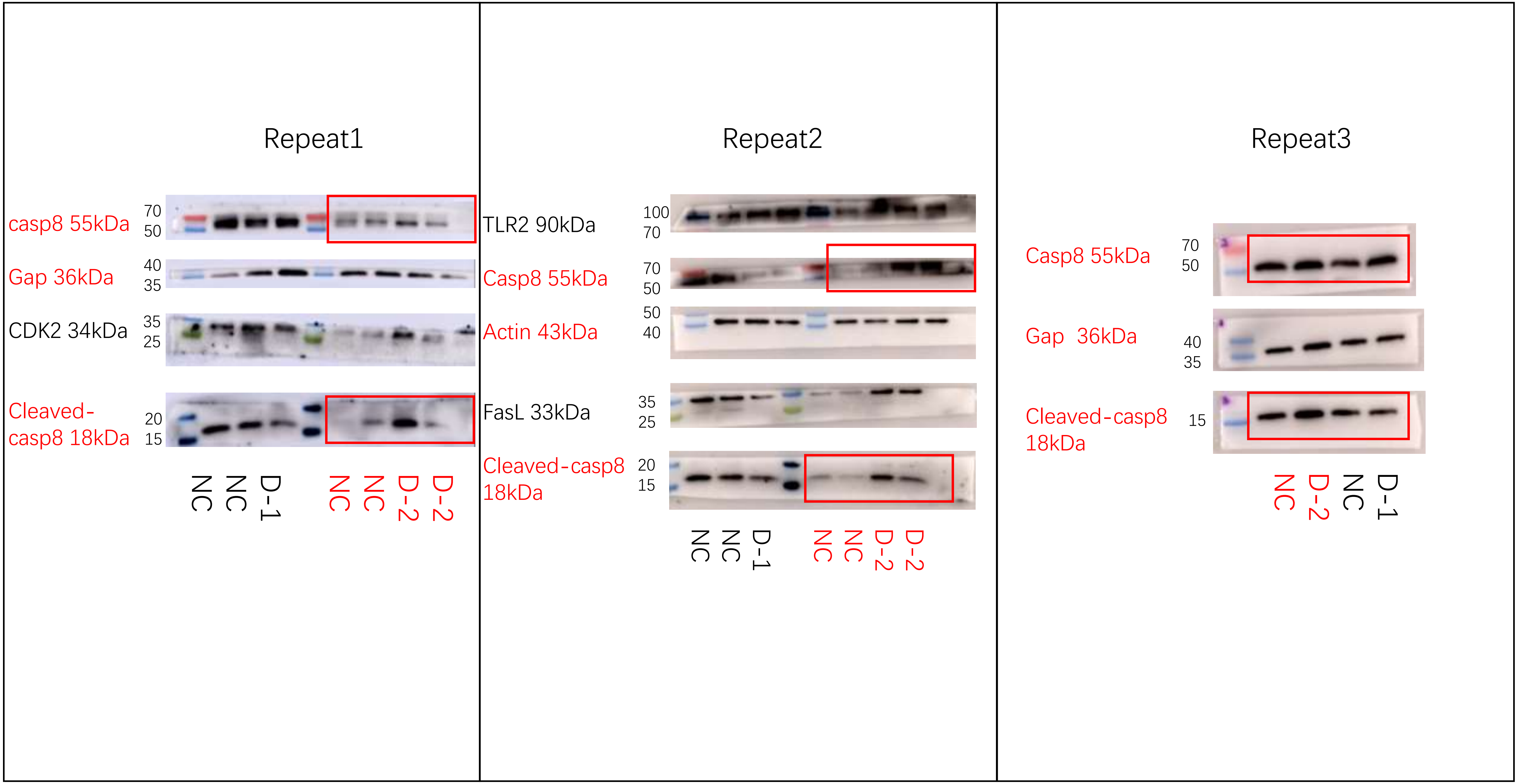

Original western blots images for Figure 4G

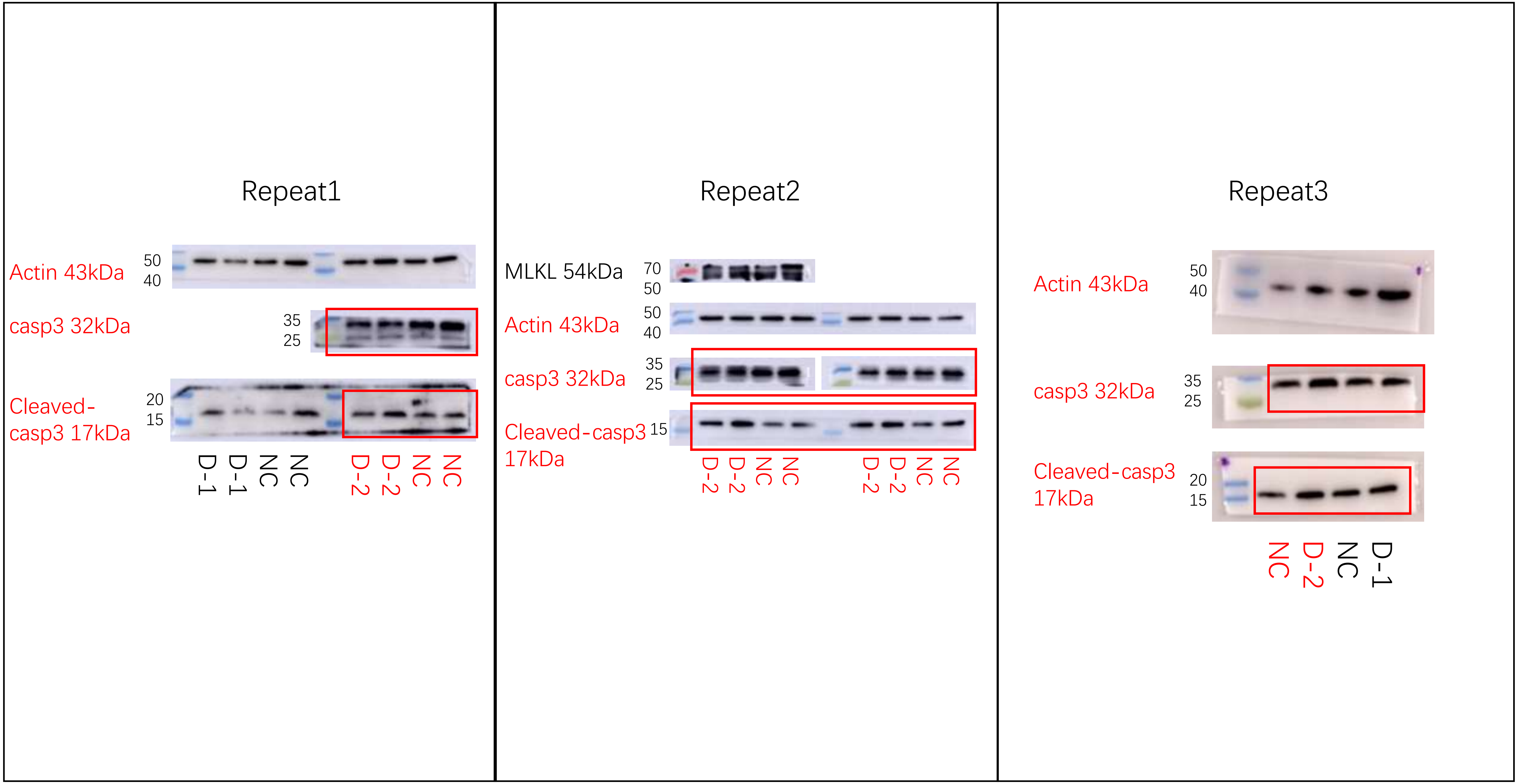

Original western blots images for Figure 5F

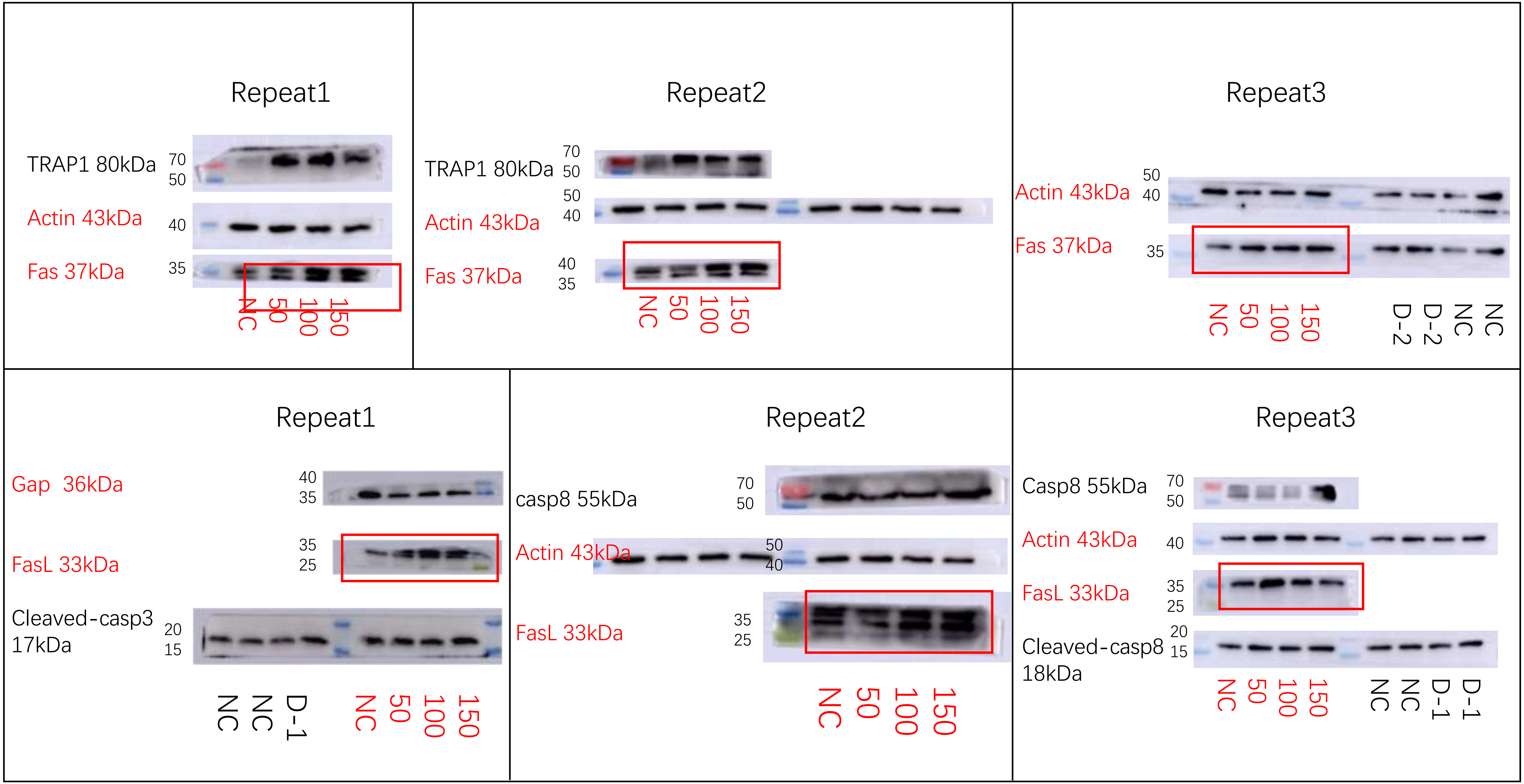

Original western blots images for Figure 5G

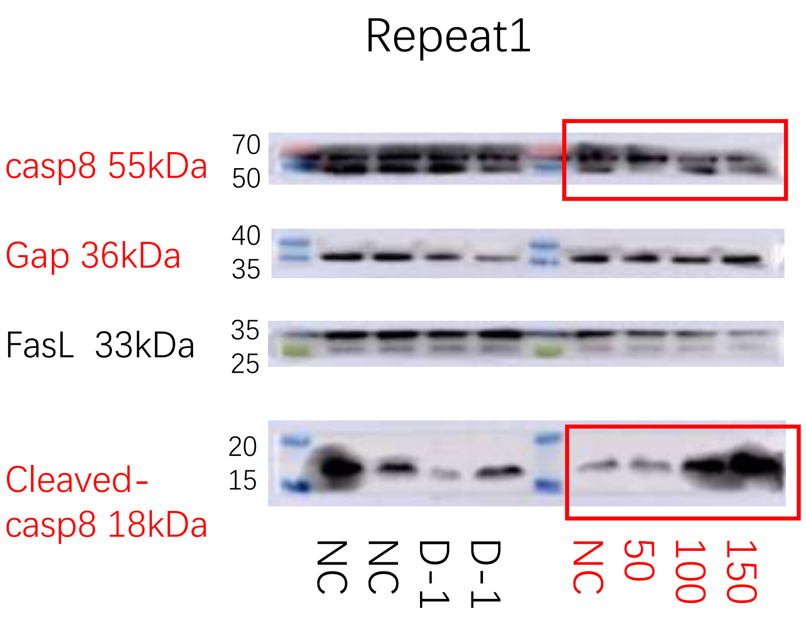

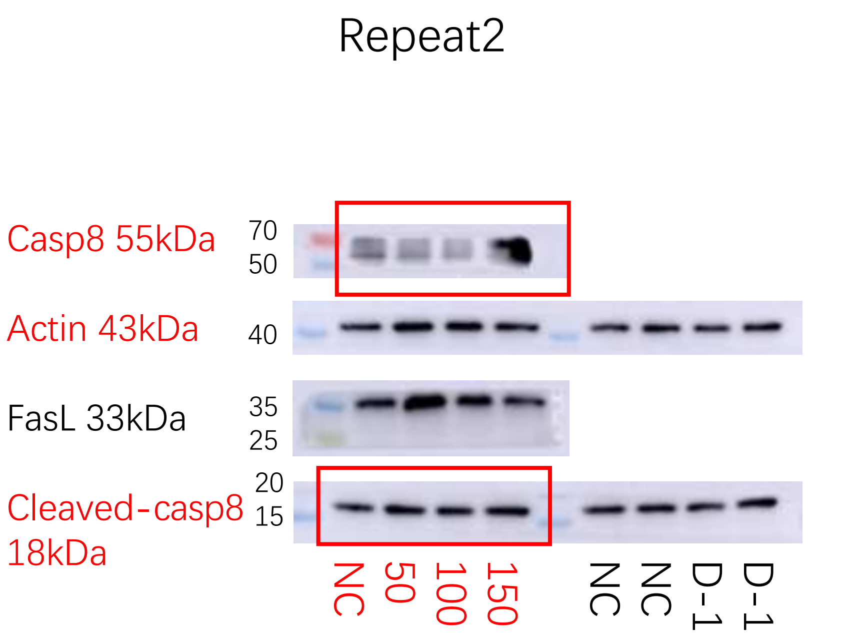

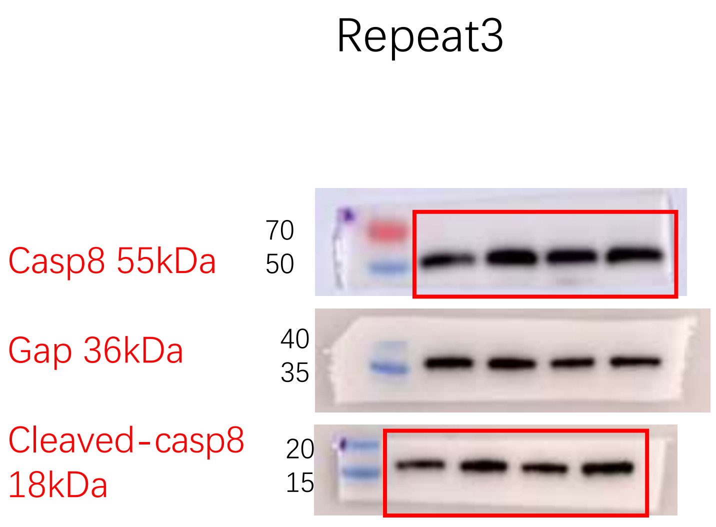

Original western blots images for Figure 5H

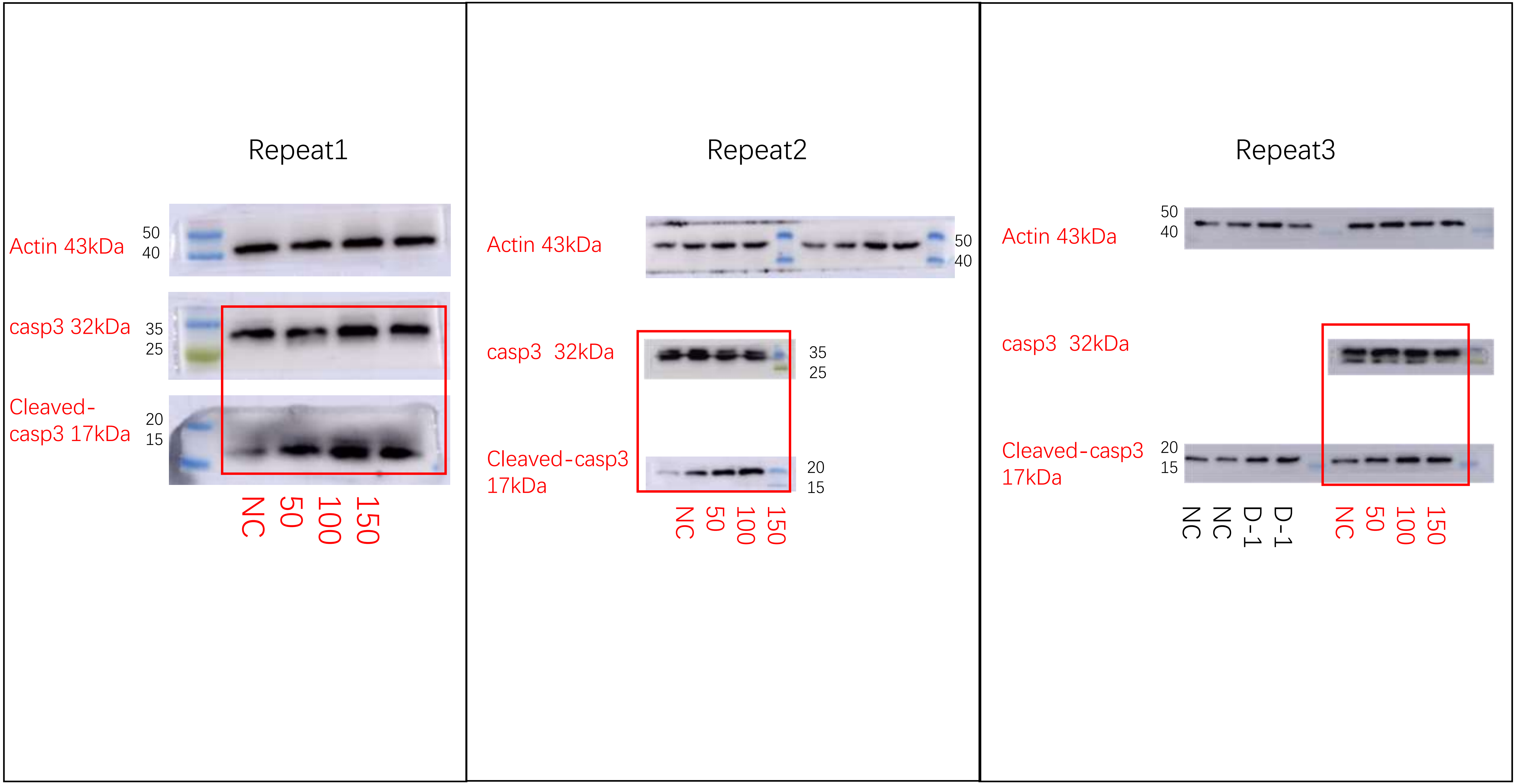

Supplement: Supplementary file 1 — Supplementary Information. [file 41598_2023_47943_MOESM1_ESM.pdf]
